# Conspicuous Response to Direct-Acting Antivirals in Chronic Hepatitis C-related Immune Thrombocytopenia: A Case Report

**DOI:** 10.7759/cureus.24193

**Published:** 2022-04-16

**Authors:** Tsung-Lung Tsai, Huei-Min Jhou, Frank S Fan

**Affiliations:** 1 Section of Gastroenterology and Hepatology, Department of Internal Medicine, Taoyuan General Hospital, Ministry of Health and Welfare, Taoyuan City, TWN; 2 Department of Nursing, Viral Hepatitis Case Management Centre, Taitung Hospital, Ministry of Health and Welfare, Taitung County, TWN; 3 Section of Hematology and Oncology, Department of Internal Medicine, Taitung Hospital, Ministry of Health and Welfare, Taitung County, TWN

**Keywords:** people who inject drugs, immune thrombocytopenia, hepatitis c virus, genotype, direct-acting antiviral

## Abstract

A 39-year-old man with a history of intravenous drug use was diagnosed to have a sudden onset of immune thrombocytopenia (ITP) in the background of a chronic hepatitis C infection with genotype 3a. Two courses of high-dose pulse dexamethasone therapy (40 mg daily for consecutive four days) failed to raise the platelet counts, but a combination direct-acting antiviral (DAA) regimen of sofosbuvir and velpatasvir, which had been proved to be effective for all hepatitis C virus (HCV) genotypes, successfully restored the platelets number to normal ranges after hepatitis C virus ribonucleic acid (RNA) was totally eliminated. Molecular mimicry of hepatitis C virus envelope proteins with platelet surface antigens is proposed to be the underlying cause of immune thrombocytopenia. An adequate direct-acting antiviral regimen is considered to be the most reliable therapy for hepatitis C-related immune thrombocytopenia.

## Introduction

Infection with hepatitis C virus (HCV) leads to the progressive development of chronic hepatitis, decompensated liver disease, cirrhosis, and hepatocellular carcinoma, causing a tremendous threat to human health. In addition to that, extrahepatic manifestations of HCV infection involve a lot of organ systems in which hematologic disorders like aplastic anemia, thrombocytopenia, and non-Hodgkin's B cell lymphoma have been reported [1]. The major causes of thrombocytopenia in HCV infection comprise bone marrow suppression, decreased production of thrombopoietin, and splenic sequestration due to hypersplenism [2]. Thanks to the rapid progress of direct-acting antivirals (DAAs) for HCV [3], rapid improvement of impaired platelet counts with DAAs in HCV-infected patients could be successfully achieved [4], especially in those with the sustained virologic response (SVR) [5]. Notably, the autoimmune process is also a mechanism of thrombocytopenia in HCV infection [6], with the cumulative incidence of immune thrombocytopenia (ITP) significantly higher in HCV-infected patients than in HCV-uninfected patients [7]. Herein, we would like to present a case of severe ITP that developed in a patient with chronic HCV infection and was found to be refractory to high-dose dexamethasone. Fortunately, dramatic regression of the ITP was observed after HCV ribonucleic acid (RNA) was eliminated by treatment with DAAs.

## Case presentation

A 39-year-old man who had been in jail for injecting drugs (methamphetamine and morphine) was brought to our outpatient clinic for evaluation of asymptomatic thrombocytopenia detected in a complete blood cell count check-up performed a few days earlier on July 13, 2021. The test revealed a white cell count of 4,500/µl (neutrophil 43.7%, lymphocyte 45.7%), haemoglobin of 14.4 g/dl, and platelet count of 36,000/µl. At the same time, also noted was mild liver function impairment with serum alanine transaminase (ALT) of 101 iu/l (reference range 5-41 iu/l) and aspartate transaminase (AST) of 74 iu/l (reference range 5-40 iu/l).

His medical record disclosed a long-existing serum anti-HCV detected for the first time 11 years ago. The status of a chronic HCV infection was confirmed by a repeated serum anti-HCV examination, and his plasma HCV RNA level turned out to be 3,990,000 iu/ml. Nevertheless, he was neither a hepatitis B virus carrier nor infected by a human immunodeficiency virus. The serum albumin (4.4 g/dl) and alfa-fetoprotein (4.42 ng/ml) were within normal limits. A sonogram of the abdomen revealed a parenchymal liver disease without focal lesions or signs of cirrhosis. His ALT and AST levels were mildly above reference ranges, but no abnormalities in thrombocyte number had been found in the past eight years. The last platelet count before this visit was 187,000/µl, checked on March 30, 2019.

Under the impression of ITP, he was treated with orally administered dexamethasone 40 mg daily for a consecutive four days as recommended in the literature [8] from July 21 to July 24, 2021. To our disappointment, no response was observed and his platelet count dropped further to 13,000/µl two weeks later on August 4. The second course of high-dose pulsatile dexamethasone was immediately prescribed, but it failed too, as the platelet count decreased to 7,000/µl two more weeks later on August 18. Although the next step of treatment could be splenectomy, rituximab infusion, or thrombopoietin-receptor agonists according to expert suggestions [9], we decided to see whether DAA therapy would be able to elevate the thrombocyte number when HCV RNA is totally eliminated.

**Figure 1:**
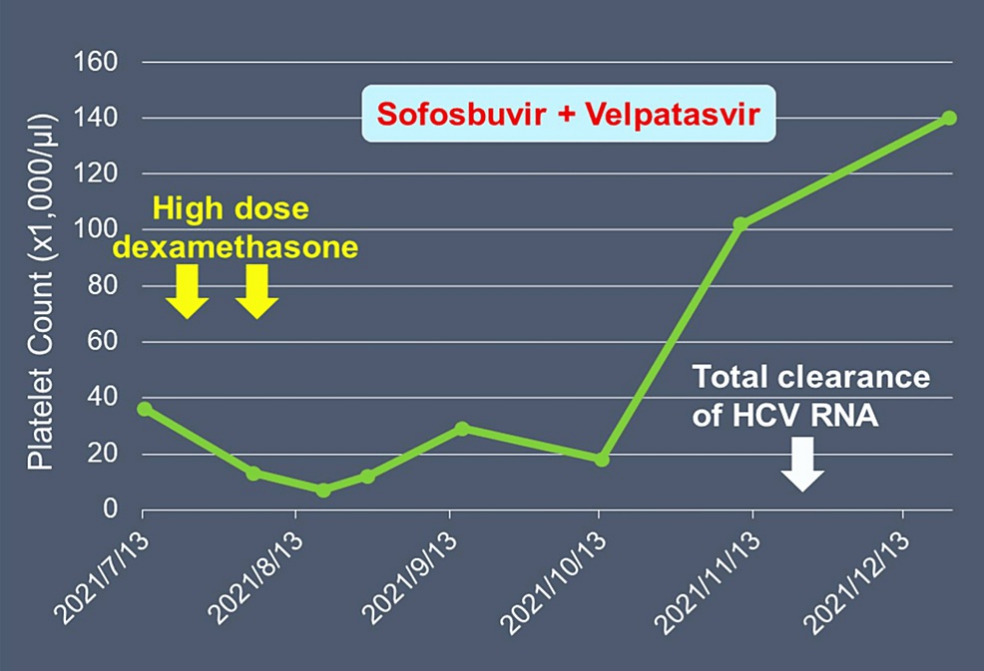
Platelet count change during the clinical course. Yellow arrows: The starting time points of the two courses of high-dose dexamethasone therapy. Light blue rectangle: The duration of direct-acting antiviral treatment. White arrow: The date when serum HCV RNA became undetectable.

The genotype of the patient’s HCV was identified to be 3a, one of the major genotypes and subtypes resulting from complex molecular evolution [10]. We chose a combination tablet (Epclusa®, Gilead Sciences Ireland UC) composing of 400 mg sofosbuvir (a non-structural 5B (NS5B) polymerase inhibitor) and 100 mg velpatasvir (a non-structural 5A (NS5A) replicase factor inhibitor) [11] as the daily regimen for a 12-week treatment course starting on August 27, 2021. This combination has been proved to be able to treat HCV infection by all genotypes [12]. As expected, the HCV RNA was quickly brought down to 27 iu/ml after treatment for four weeks on September 28 and was no longer detectable four days after completing the 12-week treatment on November 23. His serum ALT (13 iu/l) and AST (22 iu/l) also came into the normal ranges at the same time. What is more exciting is the platelet count, which suddenly jumped to 102,000/µl on day 75 of treatment (November 10) and rose to 140,000/µl 33 days after completing the whole DAA treatment course (December 22), as shown in Figure 1.

## Discussion

The global HCV prevalence is estimated at 2.5%, with genotypes 1 (49.1%) and 3 (17.9%) being the two most common strains. On the other hand, although the HCV prevalence is 4.4% in Taiwan, genotype 3 is very rare here [13]. The presence of a genotype 3a in this patient is thus an interesting finding to us. The fact that the patient belongs to a population who inject drugs (PWID), a population at high risk for HCV infection, and that genotypes 1a and 3a circulate predominantly in PWID worldwide [14], might provide a reasonable assumption for the existence of this locally rare subtype in this particular patient. That is, genotype 3a probably exists in a certain group of PWID in Taiwan, awaiting clarification after further investigation in the future.

HCV genotype 3 is a unique disease entity associated with faster progression to steatosis, cirrhosis, and hepatocellular carcinoma. Its cure rates with the first-generation DAAs, telaprevir and boceprevir, lagged behind those of other major genotypes in the past [15]. Nonetheless, the 12-week sofosbuvir-velpatasvir protocol adopted for this patient has been shown to have SVR of up to 95% in Western countries and 91% in China, respectively, for HCV genotype 3a [16], making it an excellent choice for treating HCV infection with this peculiar subtype.

It has been proposed that molecular mimicry of HCV core envelope 1 with platelet surface integrin glycoprotein IIIa (GPIIIa) can induce autoantibodies against an epitope on GPIIIa, leading to autoimmune thrombocytopenia in HCV-infected patients [17]. Antiviral treatment for HCV infection is therefore theoretically capable of curing HCV-related ITP when HCV particles are totally eliminated. The evidence supporting this proposal comes from previously published case reports. Resolution of ITP was surprisingly seen in a genotype 3a HCV infection treated by pegylated-interferon alpha 2a plus ribavirin [18], a genotype 1a HCV infection treated by sofosbuvir-ledipasvir [19], and a genotype 2a HCV infection treated by sofosbuvir-ribavirin [20]. In these three reports, standard therapies, including corticosteroids, thrombopoietin receptor agonists, and intravenous immunoglobulin for ITP, were not always successful. The case presented in this article definitely provides more confidence that regression of HCV-related ITP would be seen when the complete virologic response to DAAs is achieved despite unsuccessful treatment with high-dose dexamethasone.

## Conclusions

Based on the current evidence from our case and the other three case reports mentioned above, we would like to suggest that HCV-related ITP, even refractory to conventional therapy such as corticosteroids, might regress if HCV infection responds well to DAAs, although large-scale clinical trials are needed for a definite conclusion. The most efficient and sustained therapy for HCV-related ITP probably ought to be DAAs rather than immunosuppressants, anti-CD20 antibodies, or thrombopoietin receptor agonists. The prerequisite for a satisfactory outcome is choosing the most adequate DAA regimen based on the target HCV genotype.
